# Serum Uric Acid and Risk of Chronic Heart Failure: A Systematic Review and Meta-Analysis

**DOI:** 10.3389/fmed.2021.785327

**Published:** 2021-12-14

**Authors:** Lina Miao, Ming Guo, Deng Pan, Pengfei Chen, Zhuhong Chen, Jie Gao, Yanqiao Yu, Dazhuo Shi, Jianpeng Du

**Affiliations:** ^1^Xiyuan Hospital, China Academy of Chinese Medical Sciences, Beijing, China; ^2^Department of Graduate School, Beijing University of Chinese Medicine, Beijing, China; ^3^Cardiovascular Diseases Center, Xiyuan Hospital, China Academy of Chinese Medical Sciences, Beijing, China

**Keywords:** chronic heart failure, serum uric acid, risk factor, systematic review, meta-analysis

## Abstract

**Objectives:** A systematic review and meta-analysis was performed to evaluate the potential prognostic role of serum uric acid (SUA) in patients with chronic heart failure (CHF).

**Methods:** The Embase, PubMed, Web of Science and Cochrane Library databases were searched up to 5 April 2021 for relevant publications. Random effects model was used to pool data. STATA15.0 software was used to perform meta-analysis. Heterogeneity was assessed using the Cochran Q statistic (significance level of *P* < 0.10) and *I*^2^ statistics (significance level of 50%).

**Results:** Ultimately, 18 publications reporting adverse events in CHF patients were included. The results indicate reveal associations between a high level of SUA and the risk of all-cause mortality (HR 2.24, 95% CI 1.49–3.37), cardiovascular mortality (HR 1.14, 95% CI 1.06–1.23), and the composite of death or cardiac events (HR 1.26, 95% CI 1.01–1.56) in CHF patients. A 1 mg/dL increase in serum uric acid led to 4% (HR 1.04, 95% CI 1.02–1.05) and 9% (HR 1.09, 95% CI 1.03–1.17) increases in the risk of all-cause mortality and the composite endpoint of death or cardiac events in CHF patients, respectively.

**Conclusion:** Serum uric acid is positively associated with the risk of adverse events in CHF patients. This study protocol has been registered at PROSPERO as CRD42021247084 (https://www.crd.york.ac.uk/PROSPERO).

**Systematic Review Registration:** https://www.crd.york.ac.uk/PROSPERO.

## Introduction

Chronic heart failure (CHF) is one of the most significant public health problems, and its prevalence is increasing as the population ages (1). CHF leads to a deteriorating 5-year survival rate and poorer quality of life (2). The high rate of repeated hospitalizations and the high cost of hospitalization have severely increased the burden on the healthcare system (3). Gender, hypertension, valvular heart disease, coronary artery disease, and obesity were identified as risk factors for heart failure (4). Despite the improvement in patient management, the mortality of heart failure remains high, which calls for the need to identify other associated risk factors. Early identification of CHF patients with poor prognosis is of great significance for the treatment of CHF.

To predict the prognosis and progression of HF, several biomarkers have been used. Biomarkers (BNP and troponin), ejection fraction (EF), NYHA, etc., provide good predictive value for HF mortality. However, in the early stages of heart failure, their ability to discriminate is still uncertain. Serum uric acid (SUA), a byproduct of purine catabolism, which is well-known as a diagnostic indicator of gout. In recent years, many epidemiological studies have reported the relationship between SUA levels and a variety of cardiovascular and cerebrovascular diseases, including heart failure (5), hypertension (6), coronary artery disease (7), etc. The importance of these links, however, remains controversial. The Framingham Heart Study group, argued that uric acid is not a risk factor for cardiovascular disease and that clinicians should rely more on classic risk factors when evaluating patients (8). Major professional associations also do not consider SUA levels as a cardiovascular risk factor (9, 10). However, with the deepening of studies, several studies have found that the relationship between SUA level and the prognosis of cardiovascular diseases should be seriously considered. A meta-analysis (11) published in the European Journal of Heart Failure in 2014 linked uric acid levels to the occurrence and poor prognosis of heart failure. Since 2014, four original studies (12–15) with large samples have been published, which makes it urgent and necessary to conduct an updated meta-analysis to evaluate the relationship between serum uric acid and chronic heart failure comprehensively. Serum uric acid is a clinically readily available and low-cost test index. If serum uric acid can assess the prognosis of chronic heart failure, it will be very important for the early detection and prediction of CHF with poor prognosis. Several newly published studies have examined the significance of serum uric acid levels in the prognosis of CHF, so a focused meta-analysis of existing studies is necessary to further determine the value of SUA levels and better guide clinical practice. This systematic review and meta-analysis was designed to evaluate the potential prognostic role of SUA in patients with CHF.

## METHODS

### Systematic Literature Search

Based on the Preferred Reporting Items for Systematic reviews and Meta-Analyses (PRISMA) guidelines, a literature review and meta-analysis of the scientific peer-reviewed literature were carried out. We searched the PubMed, Web of Science, Embase, and Cochrane Library databases from the establishment of the database to 5 April 2021. The language was limited to English. The key search words used were (“left-sided heart failure” or “heart failure” or “congestive heart failure” or “cardiac failure” or “chronic heart failure”) and “urate” or “uric acid” or “hyperuricemia”). Supplementary document (Data Sheet 1) describes the search strategies used in detail. Relevant publications found in the references were also included. Gray literature (unpublished literature) and conference abstracts were not included. All included references were imported into EndNote X8, and duplicate articles were filtered and deleted. Two independent reviewers independently screened and selected the literature. In the first round of screening, studies on irrelevant topics were excluded after reading the titles and abstracts. Next, full texts were examined to exclude ineligible articles.

### Inclusion and Exclusion Criteria

The inclusion criteria were as follows: 1. observational studies or *post-hoc* analyses of randomized controlled trials; 2. studies reporting the prognostic value of adverse outcomes between SUA levels and CHF; 3. multivariate adjusted hazard ratios (HRs) with corresponding 95% confidence intervals (CIs) were published; 4. follow-up time more than 3 months. The exclusion criteria were as follows: 1. enrolment of AHF patients; 2. trial subjects were the same and did not report longer follow-up times; 3. letters, editorials, and review articles. 4. Studies of drug trials.

### Data Extraction

The following data were extracted by two reviewers independently: first author's name, sample size, design, location of study, year of publication, baseline patient profiles, SUA cutoff value, event number, follow-up duration, and unadjusted or multivariate adjusted effect sizes [HRs] for outcomes.

### Quality Evaluation

Quality assessments were independently conducted by two authors. Dispute was settled by further discussion. Should the dispute continue, a third party would be invited to the discussion for settlement. The Newcastle-Ottawa scale was used to assess quality of observational studies and cohort studies (16). The scores ≥7 points were considered as high quality.

### Statistical Analysis

We used STATA15.0 software to perform meta-analysis. The definitions of “high” and “low” serum uric acid in the included studies. For the comparison between high and low SUA levels or per 1 mg/ml SUA increase, the most fully adjusted risk was used for analysis. Heterogeneity was assessed using the Cochran Q statistic (significance level of *P* < 0.10) and *I*^2^ statistics (significance level of 50%). All combined effect sizes were analyzed by using the random effects model. Meta-regression analysis and subgroup analyses of the primary outcome were performed according to publication year, patient age, sample size, whether to adjust the confounding, quality score, and follow-up time. The subgroup differences were tested by the *Z* test (significance level at *p* < 0.05). When the number of analyzed studies exceeded 10, Begg's test (17) was used to assess publication bias. Publication bias will be adjusted by the method of trim and filling. To investigate the impact of any one study on the pooling summary, a sensitivity analysis was performed by deleting a single study in each round when the number of studies was 4 or more.

## Results

### Literature Search

**Figure 1:**
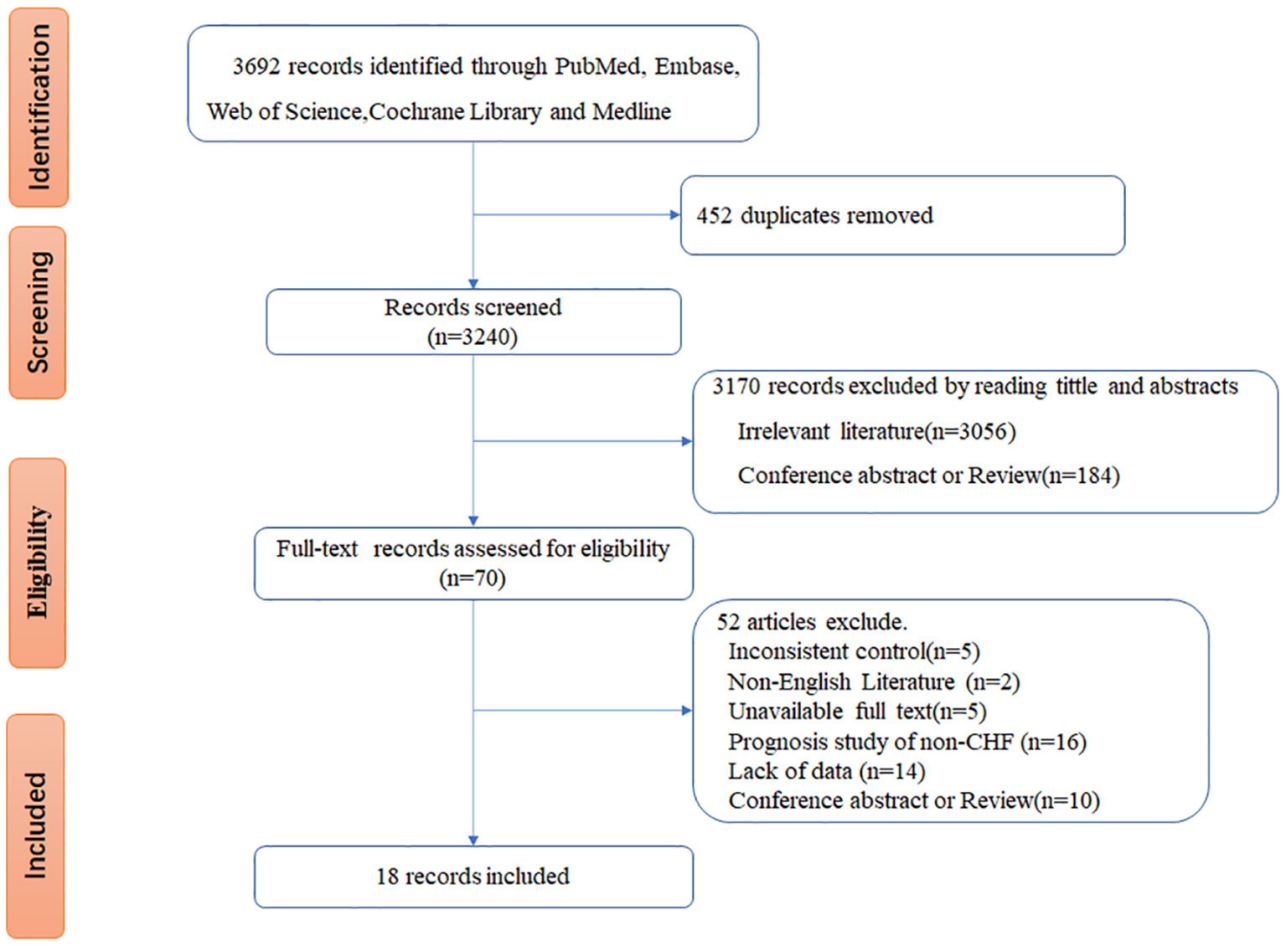
The process of study selection.

Figure 1 displays the study selection process. In total, 3,692 records were initially identified for screening from the PubMed, Embase, Web of Science, and Cochrane Library databases. By filtering the titles and abstracts, 452 duplicate articles were excluded, and 3,170 records were excluded by reading the titles and abstracts (3,056 studies because of unrelated topics and 184 because of review or conference abstracts). A full-text review of 70 studies was conducted to determine the eligible studies. Fifty-two studies were further removed for the following reasons: inconsistent control (*n* = 5), non-English literature (*n* = 2), unavailable full text (*n* = 5), prognosis study of non-CHF (*n* = 16), lack of data (*n* = 14), conference abstract and review (*n* = 10). Thus, 18 studies (12–15, 18–31) were included for further analysis.

### Study Characteristics

In Table 1, a description of baseline characteristics from the included studies is presented. A total of 32,303 patients were identified and analyzed. The mean/median age of the patients was 48 years and older. The sample sizes vary from 108 to 6,859. Follow-up time ranged from 1 to 5 years. These studies were conducted in Germany (22), Japan (19, 28, 29), Poland (14, 21, 27), the Czech Republic (31). Slovenia (26), South Korea (30), USA and Canada (20), the UK (23), Israel (18), Italy (12, 13, 24), additionally, there were two international multicenter studies (15, 25). Among them, 14 were cohort studies and 4 were case control studies. Of the 18 studies, 5 studies (14, 15, 18, 24, 31) diagnosed CHF according to the European Society of Cardiology (ESC) guidelines, while the rest studies diagnosed CHF according to clinical symptoms, physical signs, and treatment response. The laboratory measurement of serum acid uric was performed referring to enzymatic method. For methodological quality assessment, the NOS scores of these studies ranged from 5 to 9 points. Twelve of the studies had a quality score above 7, and the overall quality of the studies was high.

**Table 1:** Characteristics of included studies on the association between serum uric acid and the adverse outcomes of CHF.

**References**	**Country**	**Study design**	**Age**	**Sample size (% male)**	**Definition of hyperuricemia**	**Follow-up (year)**	**Adjusted variables**	**Outcome**	**Quality score**
Anker (22)	Germany	Cohort	59	294(85%)	10	4.3	Unadjusted	All-cause morality	6
Niizeki (28)	Japan	Case-control	77	123(48%)	7	1.2	Creatinine, NYHA functional class	Cardiac deaths and readmission	8
Jankowska (21)	Poland	Cohort	64	119(74%)	6.5	1.6	NYHA class, CrCl	All-cause morality	7
Koyama (29)	Japan	Case-control	66	141(62.4%)	Continuous	1.3	Pentosidine, BNP, eGFR, NYHA, creatinine, age, LV mass, ESV	Cardiac death, rehospitalization	7
Naruszewicz (27)	Poland	Cohort	66	108(72%)	Continuous	2.3	Unadjusted	All-cause mortality	5
Jindrich (31)	Czech Republic	Cohort	63.7	292(51%)	Continuous	5	Unadjusted	All-cause mortality	6
Lainscak (26)	Slovenia	Cohort	73	638(48%)	Continuous	2.9	COPD, age, sex, Hb, treatment with beta-blockers and furosemide	All-cause mortality	8
Kim (30)	South Korea	Case-control	61	122(62.3%)	8.7	2	Log NT-proBNP, SBP, EF, Hb, eGFR, PASP	Composite of cardiac death and readmission	6
Filippatos (20)	USA and Canada	Cohort	61.5	630(19%)	Men, 8; women, 6	2.1	Propensity score	All-cause mortality, HF hospitalization, Cardiovascular mortality	6
Hamaguchi (19)	Japan	Cohort	71.7	1,620 (60%)	7.4	2.1	Demographics, medical history, CABG, NYHA functional class, eGFR, BNP, LVEF, and medication use	All-cause death, Cardiac death, Rehospitalization, all-cause death or rehospitalization	7
Manzano (25)	Multicenter, international trial	cohort	≥70	2,128 (63%)	Continuous	1.8	Demographics, clinical, hemodynamics, laboratory, medical history, medications	Composite of all-cause mortality or cardiovascular hospital admission	7
Herrmann (23)	UK	Case–control	63	114	Continuous	1	sTNF-R1, NYHA class, cholesterol	All-cause mortality	7
Gotsman (18)	Israel	Cohort	75	6,204 (50%)	7.7	1.4	Age, sex, IHD, hypertension, AF, BMI, Hb, sodium, eGFR, urea, HF drug therapies	Mortality, Cardiac-related hospitalization	8
Baldasseroni (24)	Italy	Cohort	63	877(76%)	Continuous	1	Adjusted variables	All-cause mortality	6
Piepoli (13)	Italy	Cohort	62.7	4,577 (81%)	Continuous	3.4	Diuretic use		7
Romuk (14)	Poland	Cohort	48	774(85.8%)	Continuous	1	All demographic, clinical, echocardiography, laboratory variables, and medication data	All-cause mortality	8
Mantovani (15)	Multicenter, international trial	cohort	67	6,683 (78.3%)	Continuous	3.9	Age, sex, BMI, heart rate, total cholesterol, triglycerides, sodium, creatinine, fibrinogen, anemia, hypertension, atrial fibrillation/FL flutter, smoking, diabetes, chronic obstructive pulmonary disease (COPD), NYHA functional class, HF etiology, LV ejection fraction, and use of ACE-inhibitors/angiotensin receptor blockers (ARBs), allopurinol, statins (open/randomized) or any type of diuretic agents	All-cause death, Cardiovascular hospitalization, All-cause death or cardiovascular hospitalization (combined endpoint)	8
Canepa (12)	Italy	Cohort	67	6,859 (78.3%)	Continuous	3.5	Age, gender, BMI, heart rate, systolic blood pressure, hemoglobin, white blood cell count, total cholesterol, uricemia, glycemia, potassium, sodium, creatinine, years of HF, NYHA class, heart gallop, atrial fibrillation at ECG,	All-cause mortality	9

### SUA and All-Cause Mortality of CHF Patients

**Figure 2:**
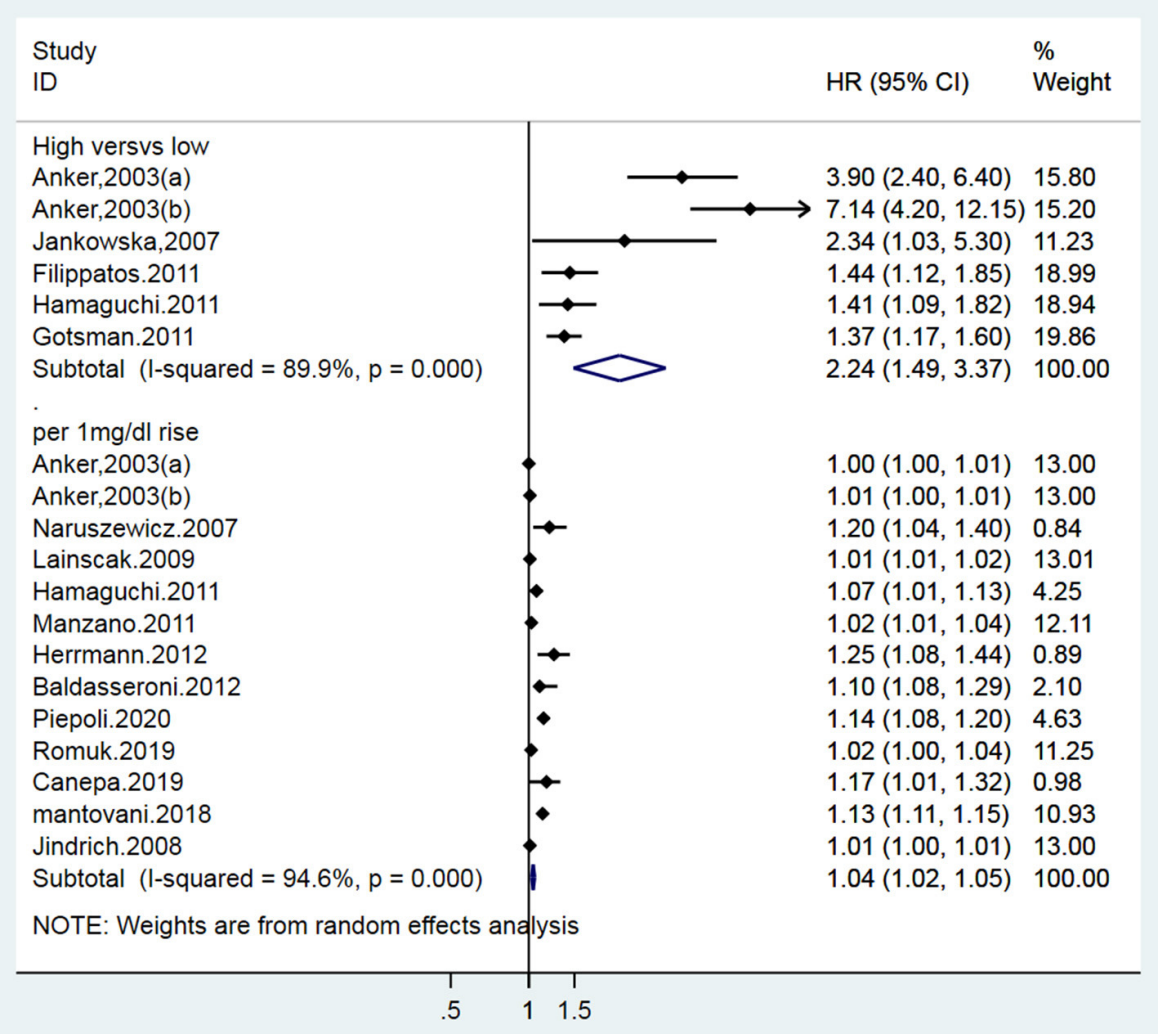
Forest plot for the association of SUA and all-cause mortality of CHF.

**Figure 3:**
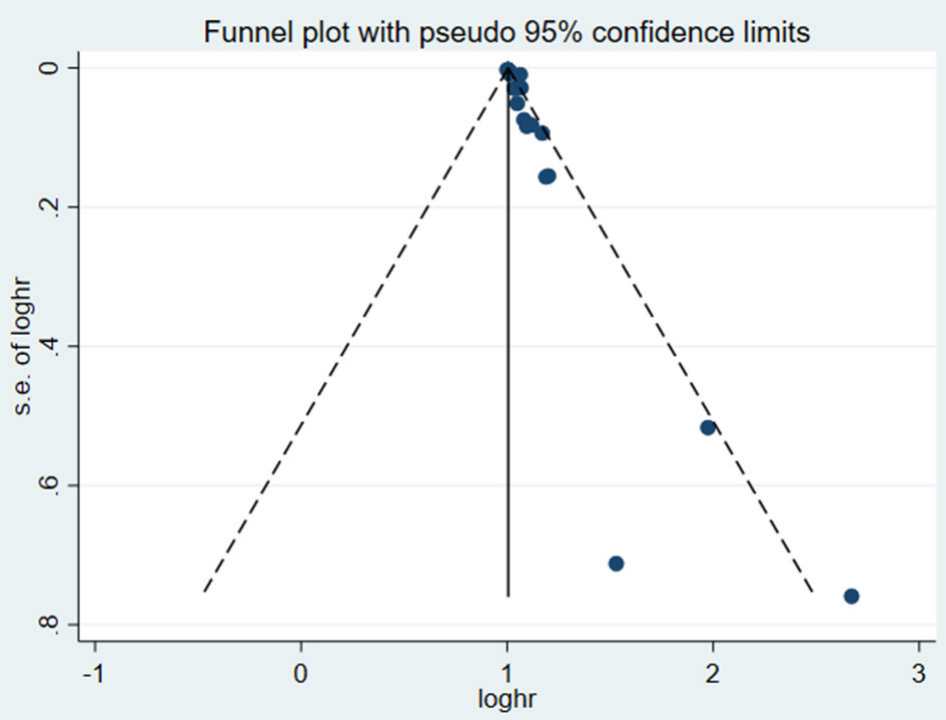
Funnel plot for the association of SUA and all-cause mortality of CHF.

**Figure 4:**
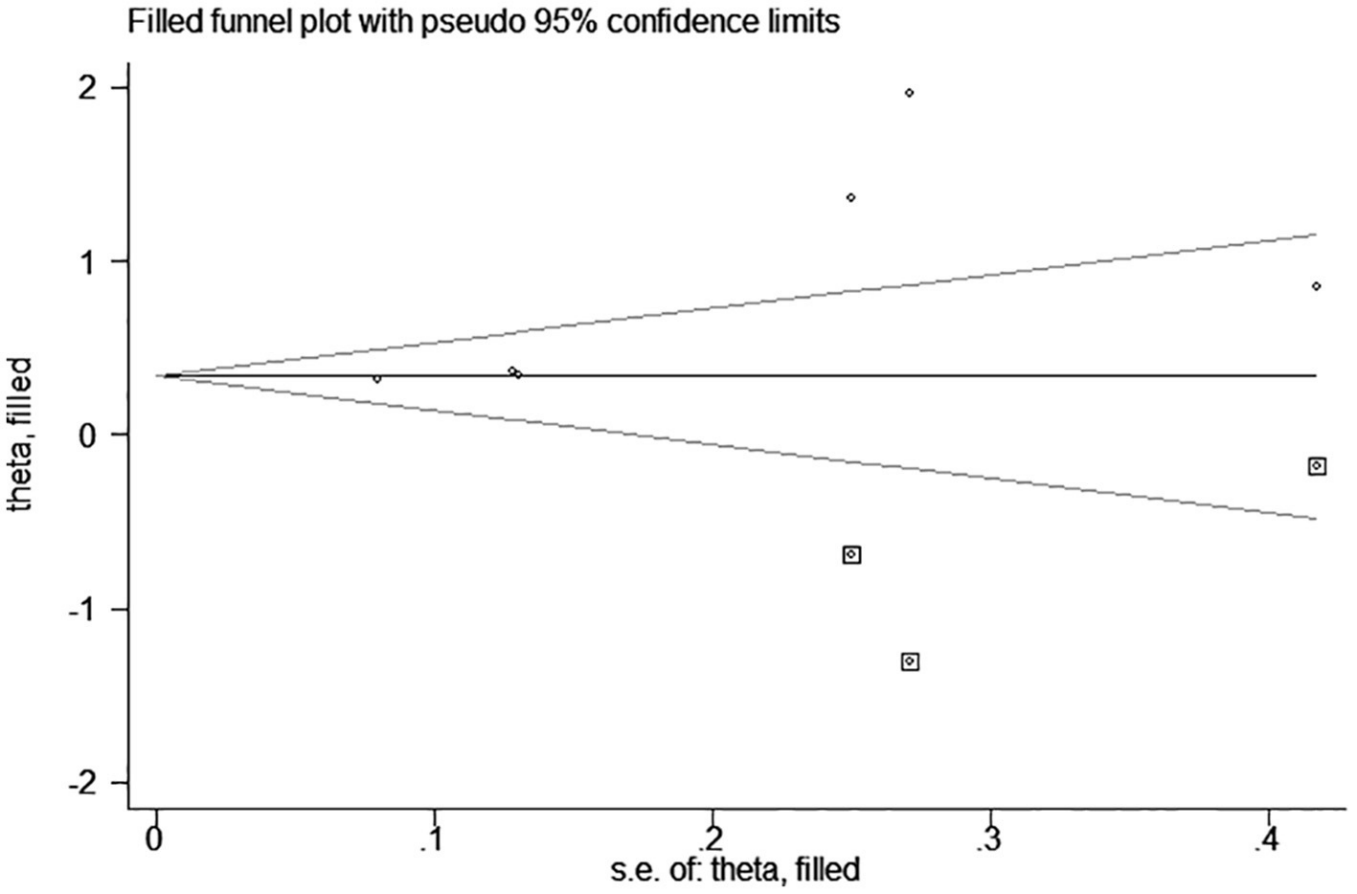
Trim and filling method analysis of SUA and all-cause mortality of CHF (categorical variables).

**Figure 5:**
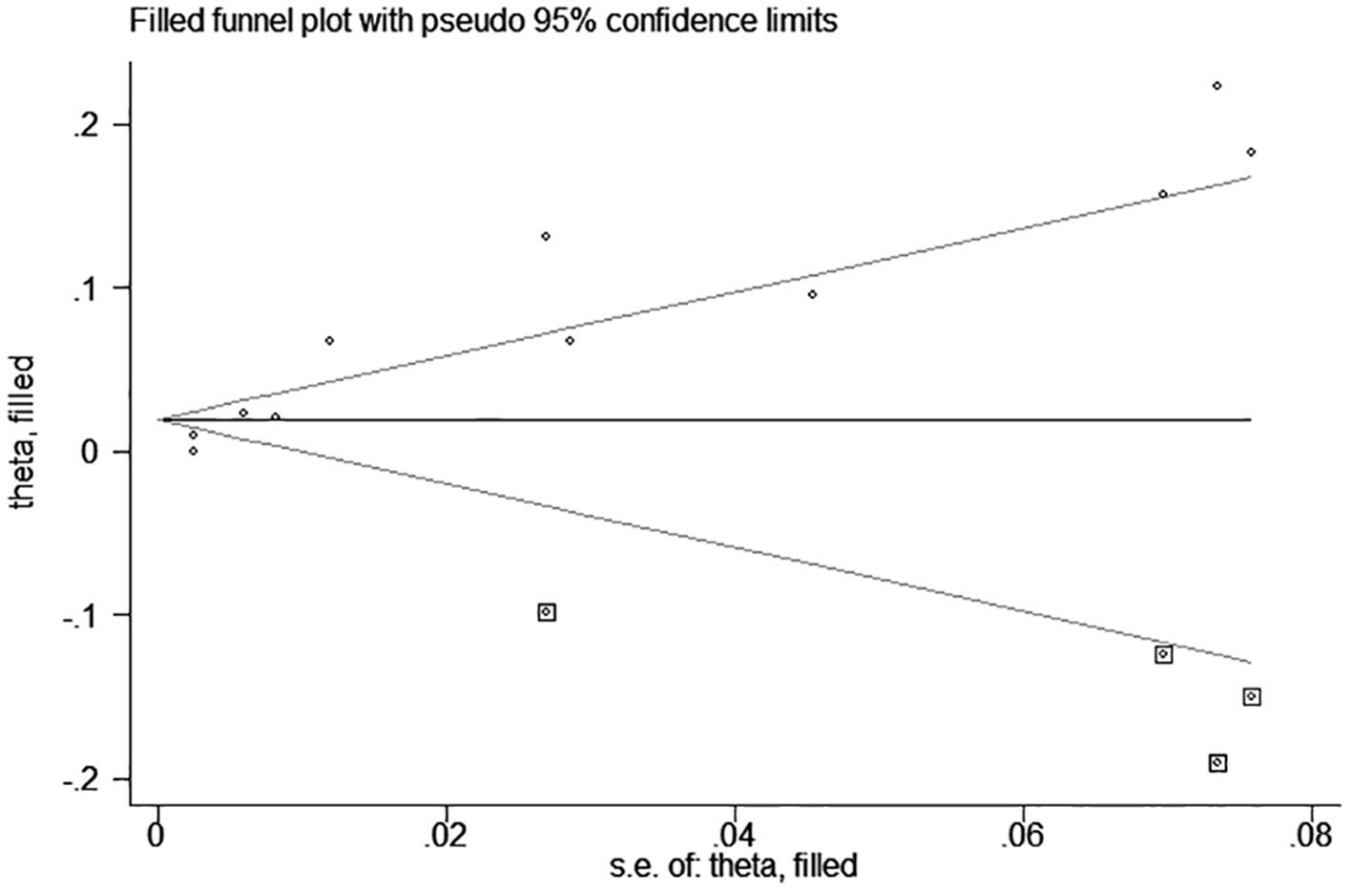
Trim and filling method analysis of SUA and All-cause mortality of CHF (continuous variables).

**Figure 6:**
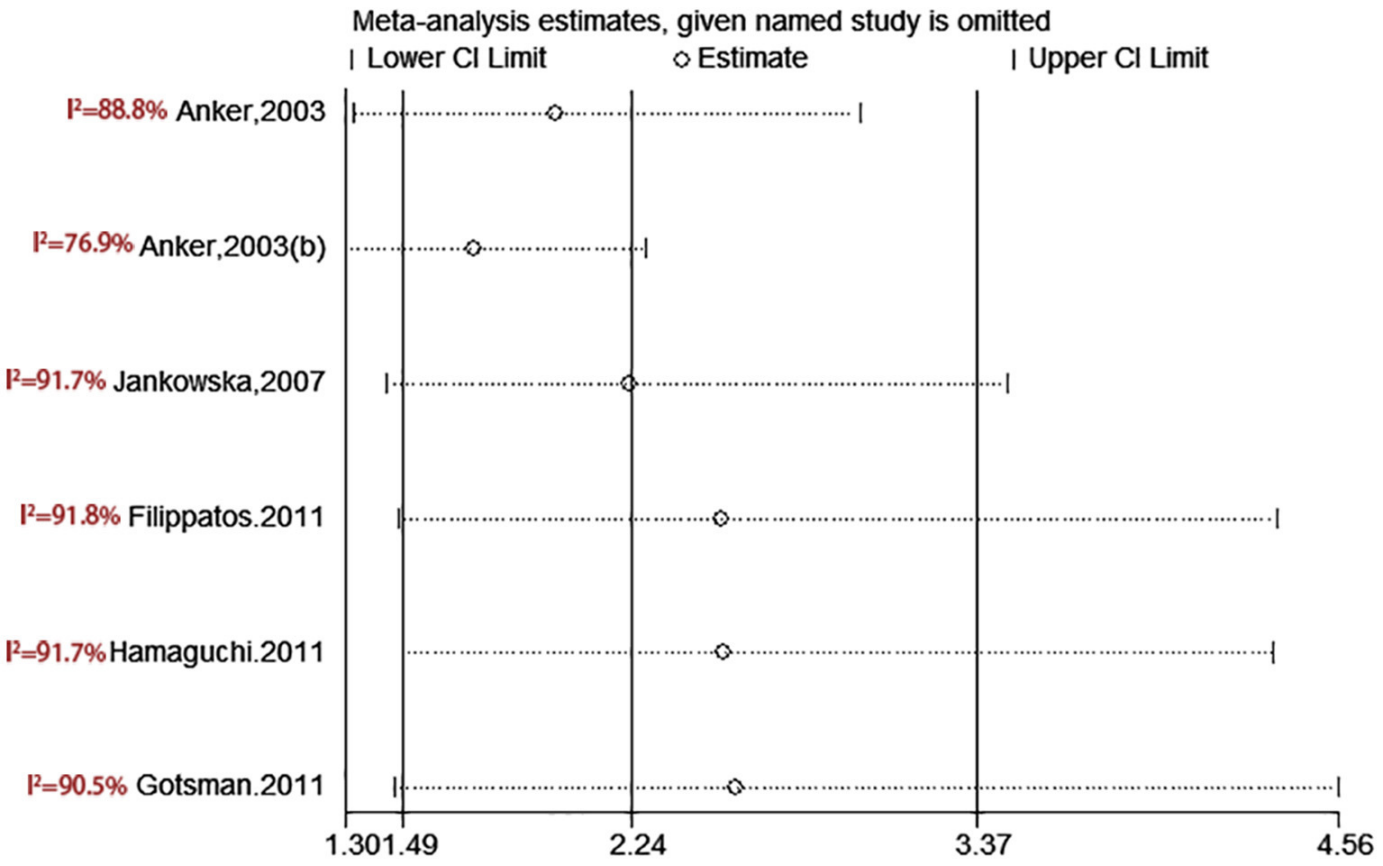
Sensitivity analysis for SUA level and all-cause mortality of CHF (categorical variables).

**Figure 7:**
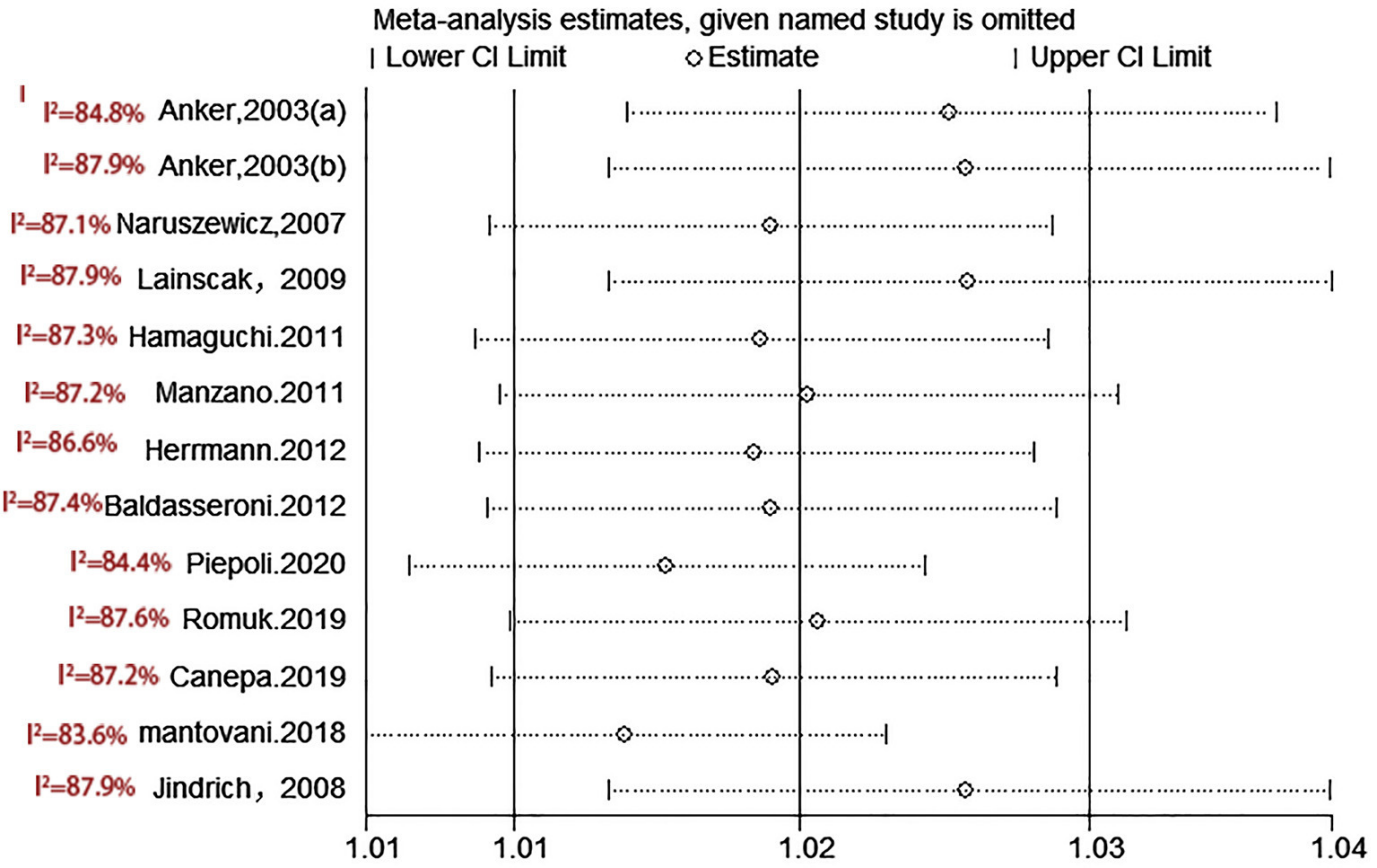
Sensitivity analysis for SUA level and all-cause mortality of CHF (continuous variables).

A total of 15 studies (12–15, 18–27, 31) (16 data sets) reported the prognostic indication of SUA for overall mortality in patients with CHF. Two of the 14 publications defined their data as both categorical and continuous variables, 5 as only categorical variables, and 12 as continuous variables. Pooled data from 5 studies (18–22) (6 data sets) with estimates as categorical variables reported the association between hyperuricemia and the risk of all-cause mortality (HR: 2.24, 95% CI: 1.49–3.37); there was appreciable statistical heterogeneity among the studies (*I*^2^ = 89.9%, *P* < 0.001) (Figure 2). Twelve studies (12–15, 19, 22–27, 31) (13 data sets) reported the estimates as continuous variables. A 1 mg/dL serum uric acid rise increased the risk of all-cause mortality by 4% (HR: 1.04, 95% CI: 1.02–1.05). Heterogeneity was observed among HRs for all-cause mortality (*I*^2^ = 94.6%) (Figure 2). Publication bias was found by Begg's

test (*P* = 0.006; *P* = 0.001) and funnel plot (Figure 3), and the trim and filling method was adopted for adjusted. The funnel plot after adjusted is shown in Figures 4, 5. The combined effect between uric acid as a categorical variable and all-cause death has changed significantly after correction (the combined effect value before trimming: HR: 2.24, 95% CI: 1.49–3.37, *P* = 0.000; after trimming: HR:1.33, 95% CI: 0.93–1.88, *P* = 0.116), but the other result is stable (the combined effect value before trimming: HR: 1.03, 95% CI: 1.02 1.04, *P* = 0.000, after trimming: HR:1.02, 95% CI: 1.01–1.04, *P* = 0.002). The results of sensitivity analysis showed that there was no significant change in the effect size when we deleted any of the studies, which indicated that our results were stable and reliable (Figures 6, 7).

### SUA and Cardiovascular Mortality of CHF Patients

**Figure 8:**
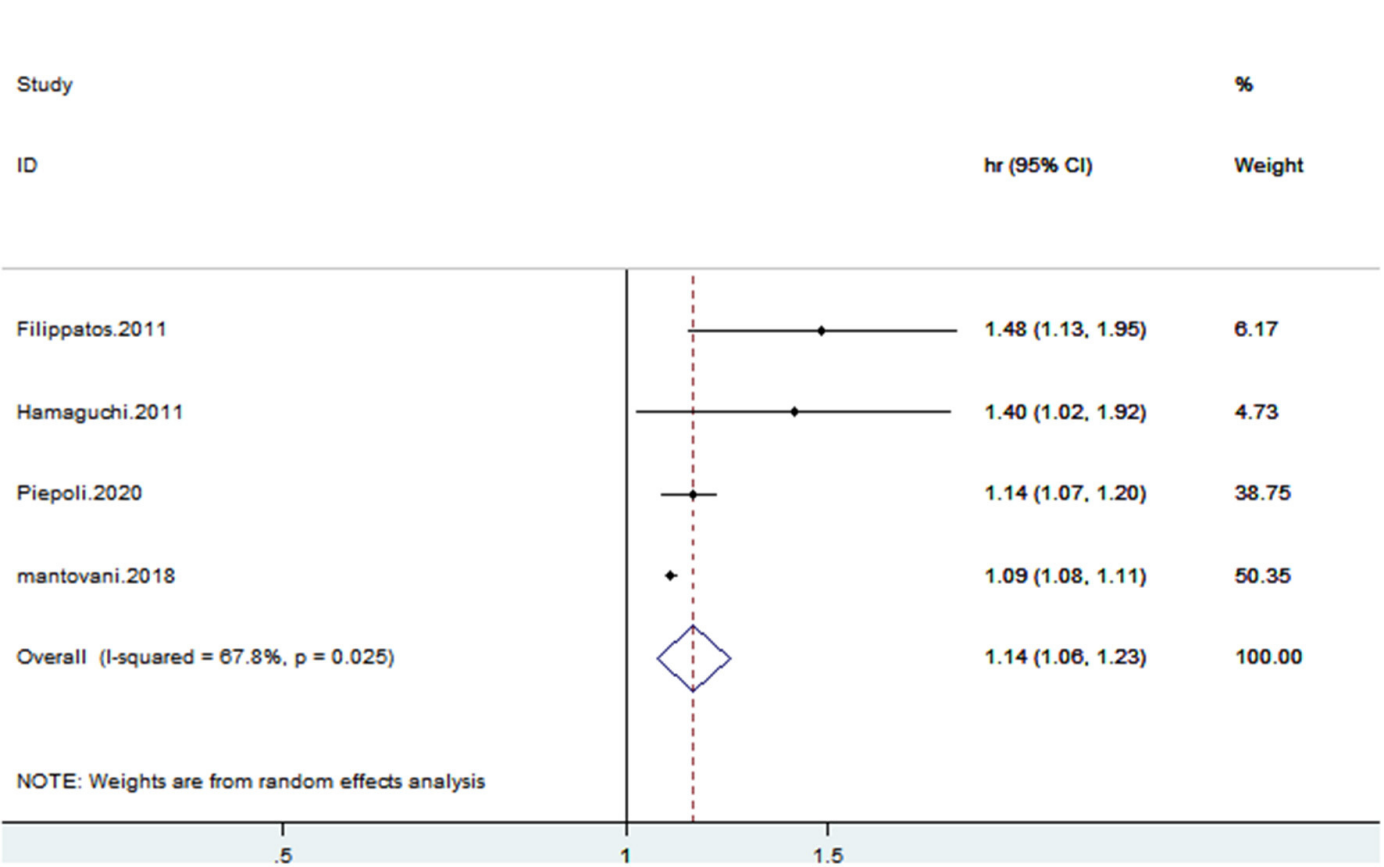
Forest plot for the association of SUA and cardiovascular mortality of CHF.

**Figure 9:**
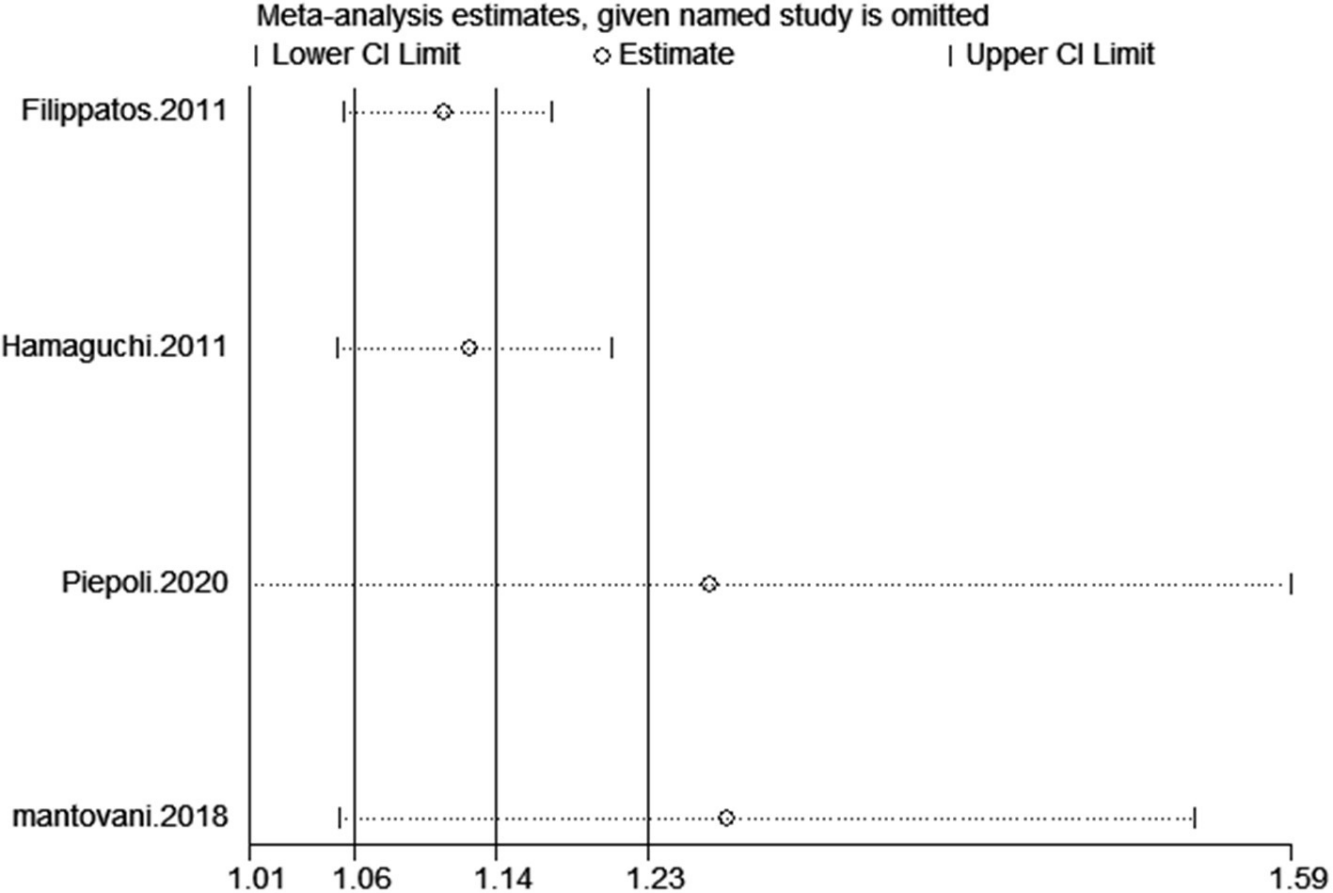
Sensitivity analysis for SUA and cardiovascular mortality of CHF.

**Figure 10:**
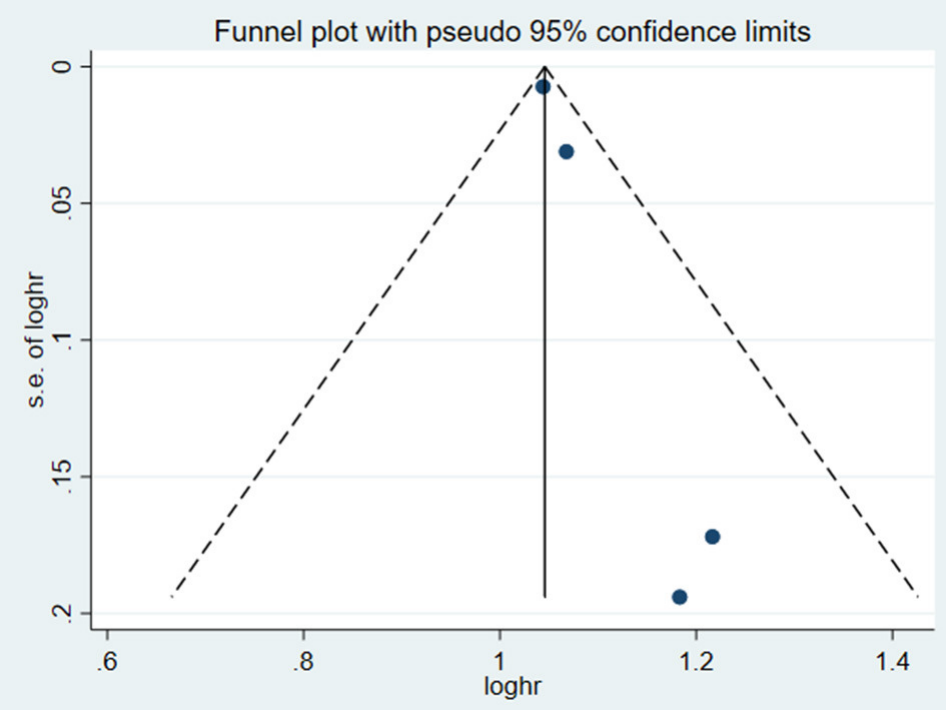
Funnel plot for the association of SUA and cardiovascular mortality of CHF patients.

Four studies (13, 15, 19, 20) reported cardiovascular mortality. CHF patients with hyperuricemia were more susceptible to cardiovascular mortality (HR: 1.14, 95% CI: 1.06–1.23); there was statistical heterogeneity among the studies (*I*^2^ = 67.8%) (Figure 8). The sensitivity analysis results were consistent (Figure 9). Publication bias was found by funnel plot (Figure 10).

### SUA and Death or Cardiac Events of CHF Patients

**Figure 11:**
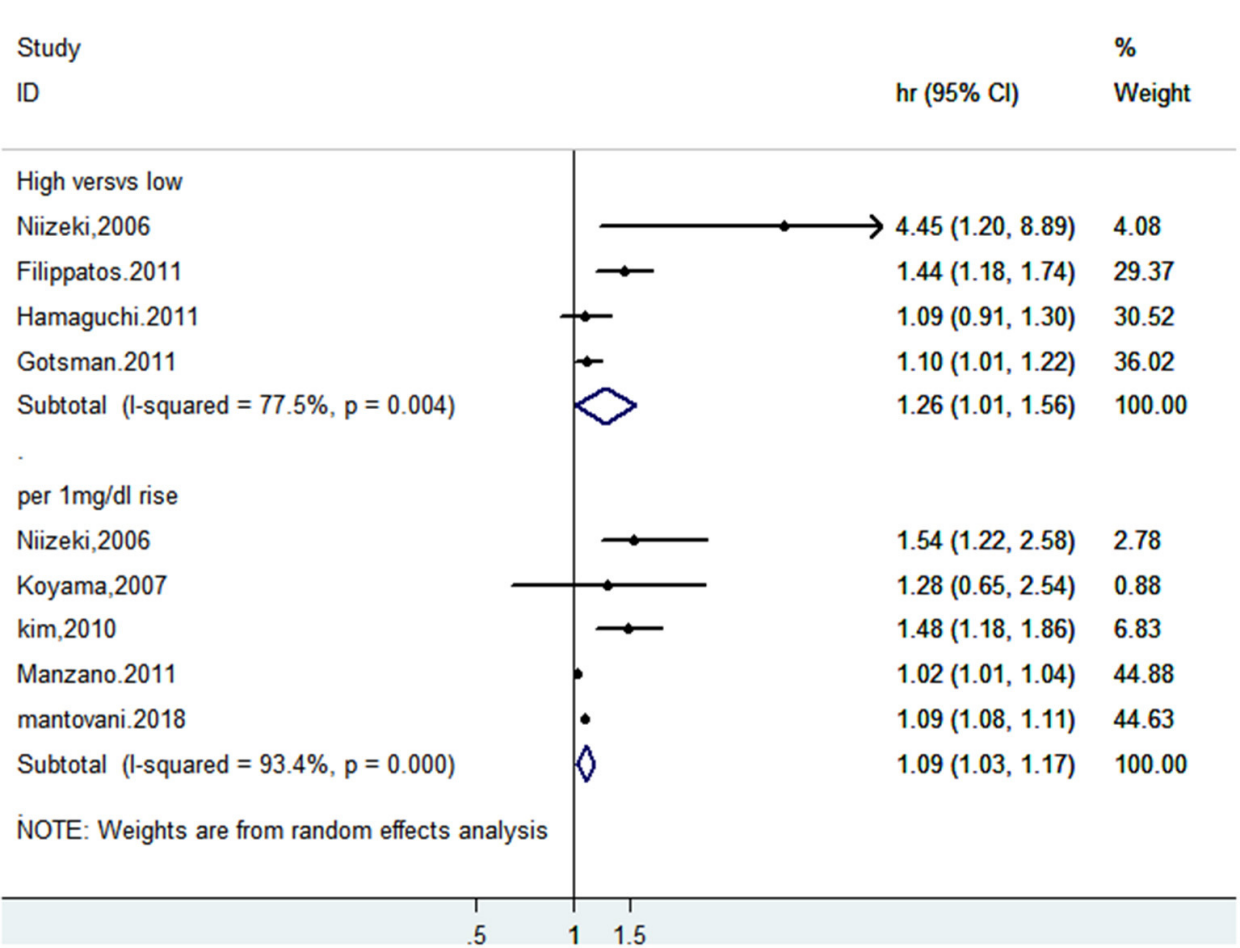
Forest plot for the association of SUA and combined death or cardiac events of CHF.

**Figure 12:**
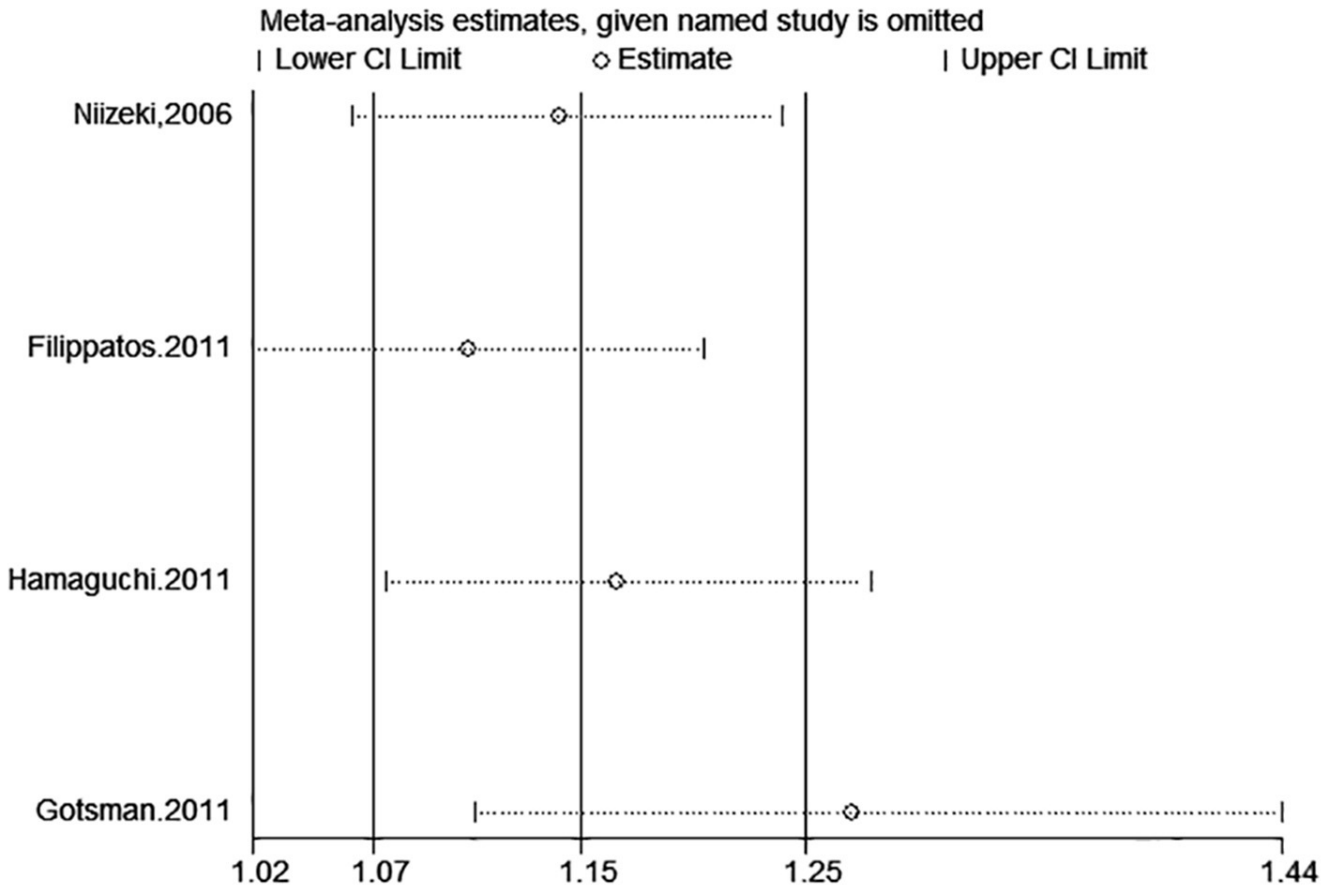
Sensitivity analysis for SUA and combined death or cardiac events of CHF (categorical variables).

**Figure 13:**
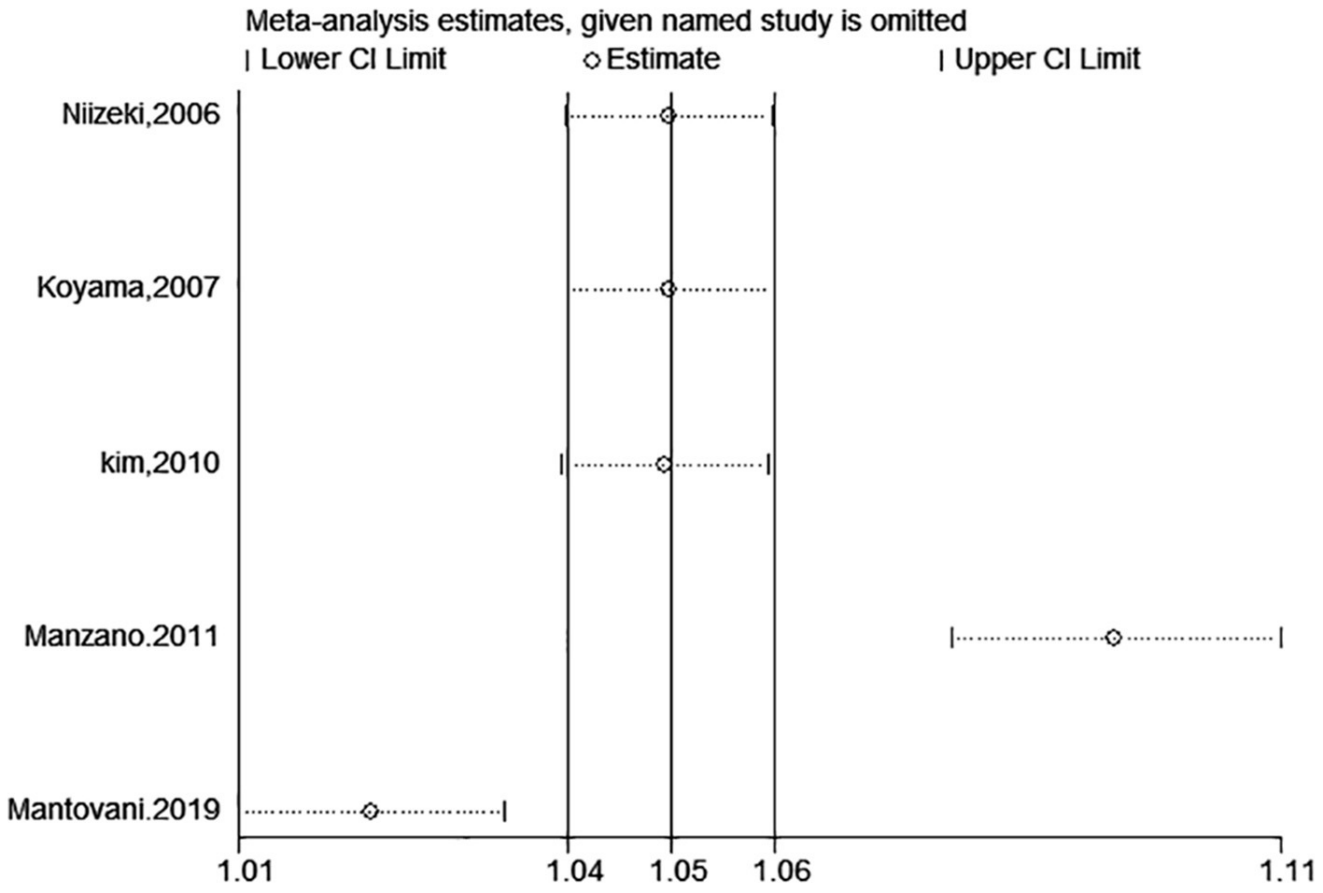
Sensitivity analysis for SUA and combined death or cardiac events of CHF (continuous variables).

**Figure 14:**
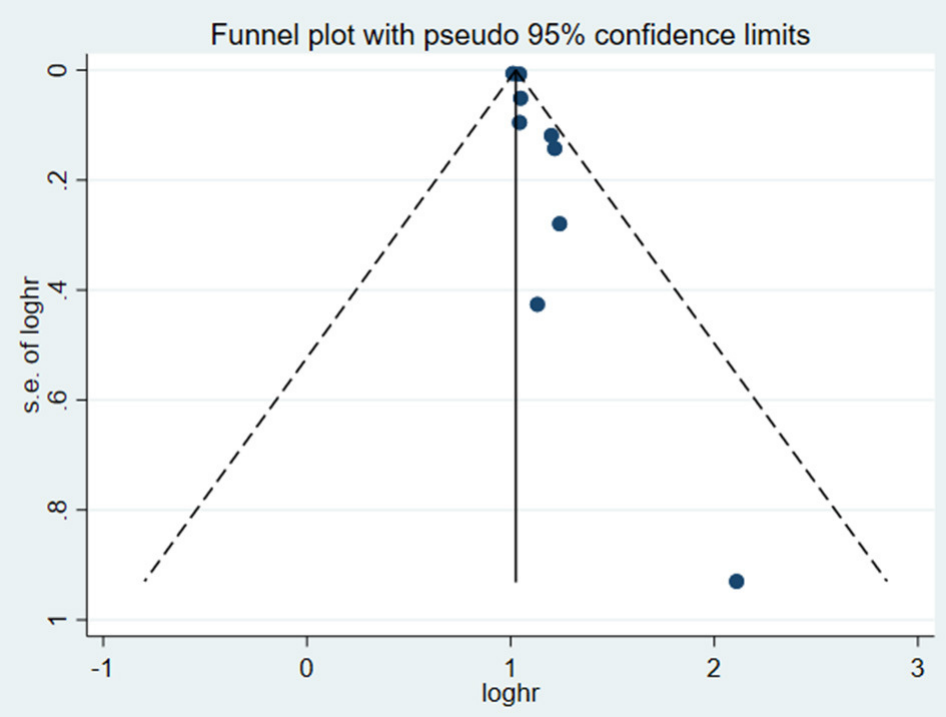
Funnel plot for the association of SUA and death or cardiac events of CHF patients.

A total of eight studies reported incidents of death or cardiac events. After pooling data from 4 studies (18–20, 28) reporting the estimates as categorical variables, hyperuricemia was linked with a growing risk of the combined incidents of death or cardiac events in CHF patients (HR 1.26, 95% CI 1.01–1.56), but substantial statistical heterogeneity was found (*I*^2^ = 77.5%) (Figure 11). A 1 mg/dL serum uric acid rise significantly increased the risk of the composite endpoint of death or cardiac events by 9% after pooling the four studies reporting the estimates as continuous variables (HR 1.09, 95% CI 1.03–1.17) (15, 25, 28–30). In addition, a significant degree of heterogeneity was present (*I*^2^ = 93.4%) (Figure 11). The results of the sensitivity analysis were consistent (Figures 12, 13). Publication bias was found by funnel plot (Figure 14).

### Subgroup Analysis

To explore the heterogeneity of the study, we conducted a subgroup analysis of all-cause mortality based on publication year, patient age, sample size, quality score, and follow-up time. The results of subgroup analysis show that none of these factors are the main sources of heterogeneity. The test of differences between subgroups shows that the effect size of the results may be affected by the sample size, follow-up time, publication year, and whether to adjust factors (Tables 2, 3).

**Table 2:** Subgroup analyses of the relationship between SUA and all-cause mortality in CHF patients (categorical variable).

**Subgroup**	**Study**	**Heterogeneity**		**Meta-analysis**		**Number of study**	***P*-value of**
		***I* ^2^**	***P*-value**	**Effect size**	**95% CI**		**Difference between subgroups**
Sample size	<500	64.8%	0.059	HR 4.26	2.39–7.62	3	0.00
	≥500	0.0%	0.941	HR 1.39	1.24–1.57	3	
Adjustment	Adjusted	0.0%	0.653	HR 1.41	1.26–1.58	4	0.00
	Unadjusted	62.8%	0.101	HR 5.23	2.89–9.46	2	
Follow-up (year)	<2	36.8%	0.208	HR 1.53	1.00–2.34	2	0.58
	≥2	92.8%	0.000	HR 2.64	1.38–5.03	4	
Publication year	After 2010	0.0%	0.941	HR 1.39	1.24–1.57	3	0.00
	Before 2010	64.8%	0.059	HR 4.26	2.39–7.62	3	
Age	<70	62.8	0.101	HR 5.23	2.89–9.46	2	0.00
	≥70	0.0%	0.653	HR 1.41	1.26–1.58	4	
Quality score	<7	86.0%	0.000	HR 1.63	1.17–2.28	3	0.53
	≥7	0%	0.934	HR 1.43	1.19–1.71	3	

**Table 3:** Subgroup analyses of the relationship between SUA and all-cause mortality in CHF patients (continuous variable).

**Subgroup**	**Study**	**Heterogeneity**		**Meta-analysis**		**Number of study**	***P*-value of difference**
		***I* ^2^**	***P*-value**	**Effect size**	**95% CI**		**between subgroups**
Sample size	<500	86.3%	0.000	HR 1.01	0.99–1.03	4	0.01
	≥500	86.1%	0.000	HR 1.03	1.02–1.05	9	
Adjustment	Adjusted	80.7%	0.000	HR 1.05	1.03–1.08	4	0.00
	Unadjusted	87.2%	0.001	HR 1.01	1.00–1.01	9	
Follow-up(year)	<2	70.1%	0.018	HR 1.03	1.01–1.06	3	0.08
	≥2	88.8%	0.000	HR 1.02	1.01–1.03	7	
Publication year	After 2010	80.5%	0.000	HR 1.05	1.03–1.08	9	0.00
	Before 2010	80.8%	0.001	HR 1.01	1.00–1.01	4	
Age	<70	77.14%	0.004	HR 1.008	1.002–1.015	4	0.45
	≥70	88.19%	0.000	HR 1.069	1.040–1.099	9	
Quality score	<7	85.3%	0.000	HR 1.03	1.01–1.05	6	0.35
	≥7	83.8%	0.000	HR 1.04	1.02–1.06	7	

### Meta-Regression Analysis

To explore the source of heterogeneity, we conducted meta-regression analysis using mean age, gender, and mean follow-up time as covariables, and the results showed that age, gender and mean follow-up time had no statistical significance (*p* > 0.05), so they were not the main sources of heterogeneity (Tables 4, 5).

**Table 4:** Meta regression analysis of SUA and All-cause mortality of CHF patients.

**Concomitant variable**	**Coef**.	**Std. Err**	**z**	***P* > |z|**	**[95% Conf. Interval]**	
Mean Age	0.0007895	0.0004926	1.6	0.109	−0.0001761	0.001755
Mean follow up time	−0.0014168	0.0017655	−0.80	0.422	−0.0048772	0.0020436
Gender	0.026645	0.0183555	1.45	0.147	−0.009331	0.0626211
_cons	0.9443494	0.0442626	21.34	0.000	0.8575963	1.031102

**Table 5:** Meta regression analysis of SUA and Death or cardiac events of CHF patients.

**Concomitant variable**	**Coef**.	**Std. Err**	**z**	***P* > |z|**	**[95% Conf. Interval]**	
Mean age	−0.0032499	0.0077723	−0.42	0.676	−0.0184834	0.0119836
Mean follow-up time	0.0411667	0.022369	1.84	0.066	−0.0026758	0.0850092
Gender	−0.4208582	0.2307228	−1.82	0.068	−0.8730667	0.0313502
_cons	1.430657	0.5598989	2.56	0.011	0.3332758	2.528039

## Discussion

Our systematic review and meta-analysis of 18 studies with over 33,000 sample sizes suggested that a high level of SUA independently predicted the risk of all-cause mortality, cardiovascular death and combined death or cardiac events in CHF patients.

Three previous meta-analyses also examined the relationship between elevated SUA levels and the occurrence and prognosis of HF. A meta-analysis (11) of 33 studies published in 2013 by Huang et al. showed that elevated SUA levels are associated with an increased risk of HF and adverse outcomes in patients with HF. Another meta-analysis (32) also identified a higher level of SUA as a strong independent predictor of all-cause mortality in patients with HF. However, the patients included in the above two studies were broadly defined. The relationship between different types of HF and SUA levels was not carefully analyzed. A meta-analysis (33) published in 2019 analyzed the relationship between serum uric acid level and the prognosis of AHF, and the results showed that the elevated of SUA level independently predicted all-cause mortality and combined endpoint death or readmission in AHF patients. However, the association between SUA levels and CHF remains unclear Kazuhide Ogino et al. used the uric acid-lowering drug benzbromarone to observe the treatment effect of 14 CHF patients with hyperuricemia. The results of the study showed that benzbromarone significantly reduced SUA but brain natriuretic peptide, left ventricular ejection fraction and the size assessed by echocardiography did not change (34). Our study analyzed all the current studies on the relationship between SUA and the prognosis of CHF, and the results provided more proof information that a higher SUA level is a comprehensive risk factor for cardiac events, cardiovascular mortality and death, and all-cause mortality in patients with CHF.

The clear pathophysiologic role of serum uric acid in CHF progression is not yet clear, it is speculated to be: (i) Functional upregulation of a key enzyme in purine metabolism, xanthine oxidase (XO), which derives reactive oxygen species that may be responsible for a series of harmful processes in the pathophysiology of CHF, such as cardiac hypertrophy, myocardial fibrosis, left ventricular remodeling and impaired contractility. (ii) Uric acid impairs vascular endothelial cells and causes poor prognosis (35). A previous randomized, placebo-controlled study reported that allopurinol can improve endothelial dysfunction in patients with CHF (36). (iii) Hyperuricemia is associated with worse hemodynamic measures (37). (iii) In patients with CHF, increased SUA levels demonstrated a significant association with diastolic dysfunction (38). In two placebo-controlled studies, allopurinol treatment improved blood flow and peripheral vasodilation in patients with CHF (39). In addition, sympathetic nerve excitement and the release of catecholamine neurotransmitters in patients with HF lead to the constriction of renal glomerular arterioles, a decrease in the glomerular filtration rate, and a decrease in uric acid excretion, thus leading to an increase in uric acid. The increase in uric acid further activates the renin-angiotensin-aldosterone system, leading to ventricular remodeling and poor prognosis in patients with HF (40). Studies have also pointed out that the serum uric acid level of patients with chronic ischemic heart failure is related to left ventricular remodeling (41).

It is worth noting that the heterogeneity of the included studies was high. The potential sources of differences are age, sample size, variable adjustment, follow-up time, literature quality score and publication year. Based on the above factors, we conducted a subgroup analysis to explain the heterogeneity, but this did not find the source of heterogeneity. However, the results of our subgroup analysis did not change the conclusion that elevated uric acid would increase the risk of all-cause death. In addition, according to the results of the subgroup analysis, we also found that the size of the effect may be affected by factors such as sample size, follow-up time, publication year, and age of the research subjects. The reason may be as follows: there is a significant difference between the research results before and after 2010. Combined with the subgroup analysis based on sample size, it can be inferred that the main reason may be related to the sample size. The sample size studied before 2010 was small, while the sample size studied after 2010 was larger. Subgroup analysis of studies with adjusted or unadjusted variables showed that unadjusted studies found that the risk of all-cause death in patients with heart failure was higher than that in adjusted studies. Although multivariate adjustments have been made in most observational studies, residual confounding factors still inevitably exist. Among them, the use of diuretics and the degree of renal damage may play a key role in variable factors. Uric acid is the final metabolite of purine and is catalyzed by xanthine oxidase. Under normal circumstances, the production and excretion of uric acid are in dynamic balance, and all factors that can affect the production or excretion of uric acid, such as the activation of xanthine oxidase, the use of diuretics, and the injury to renal function, can affect the concentration of uric acid in the body. Diuretics are the basic drugs for the treatment of HF. The extensive use of diuretics can not only increase uric acid, but also produce many adverse reactions, such as electrolyte disturbance, hemodynamic instability, and neuroendocrine activation. These adverse reactions can increase the risk of death and lead to poorer prognosis in patients with heart failure. Other factors that influence uric acid excretion, such as reduced glomerular filtration rate, hyperinsulinemia, renal vasoconstriction, and factors that increase uric acid production, such as tissue ischemia or oxidative stress, may influence the prognosis of uric acid and chronic heart failure.

Although there have been many studies on the relationship between uric acid and cardiovascular disease, a crucial question about how to determine the cut-off of cardiovascular uric acid needs to be answered. The actual cut-off of hyperuricemia (>6 mg/dL in women and 7 mg/dL in men) is mainly based on the saturation point of uric acid, but previous evidence suggests that low levels of uric acid may also have a negative impact on the cardiovascular system. The recently published URRAH study reported the uric acid cut-off for total and cardiovascular mortality, which indicated that the optimal cut point for total mortality and cardiovascular mortality was 4.7 and 5.6 mg/dL, respectively (41). This research also proposes that the cut-off of uric acid may be different for various disease outcomes. For example, the research mentioned that uric acid cut-off for fatal myocardial Infarction is 5.70 mg/dL (41). Currently, a little data can be used to back up the critical point of uric acid for CHF, and our study merely incorporates the current mainstream uric acid critical point research. Thus, more studies are needed in the future to further determine the critical point of uric acid that affects the prognosis of CHF, but our current study has already confirmed that the high uric acid will lead to the poor prognosis of CHF. In addition, attention should be paid to the influence of age and sex on the prognosis of uric acid and CHF. The study by Eugenio R. Cosentino et al. demonstrated that serum uric acid in male elderly patients with heart failure seems to be negatively correlated with EF%, and SUA has a severe effect on left ventricular function (41). A study from Norway also found that a high level of SUA was independently associated with lower 5-year survival rates of outpatient women rather than men (42). The Brisighella study revealed that vascular damage associated with high levels of SUA is age-dependent and reversible (43). Thus, stratification factors, including patients' population, age, sex, as well as the critical value of uric acid, need to be taken into consideration to better interpret serum uric acid and its correlation with CHF.

### Strength and Limitation

To the best of our knowledge, this is the first meta-analysis focusing on the relationship between uric acid level and the prognosis of CHF. Our meta-analysis showed that uric acid level is an independent predictor of all-cause death, cardiovascular death and the composite end point of death or cardiovascular events in CHF patients. Moreover, included studies from different countries were included, and the sample size was large, so the results were relatively stable and reliable. All the quality of the included studies was relatively high. Although there was heterogeneity in the results, the results of our subgroup analysis and sensitivity analysis were stable.

However, our study also has limitations. First, the heterogeneity in our study was significant. Even though the sensitivity analysis and subgroup analysis were adopted, we still failed to explore the origin of heterogeneity. Second, the lack of repeated measurements of serum uric acid levels was a common limitation in the included studies. One time determination of serum uric acid levels may not accurately reflect the changes with time. Third, individual studies have different threshold for high and low uric acid, which would have an impact on our research results and may be a reason for the heterogeneity of outcome indicators. Four, considering the quality of the included research, we did not include some conference studies and gray literatures with small sample sizes, which may cause the publication bias. Finally, due to the insufficient of data, the relationship between serum uric acid levels and different stages of chronic heart failure cannot be evaluated in our meta-analysis.

### Conclusion

High SUA levels independently predicted the risk of all-cause mortality, cardiovascular death and combined death or cardiac events in CHF patients.

## Data Availability Statement

The original contributions presented in the study are included in the article/Supplementary Material, further inquiries can be directed to the corresponding author/s.

## Author Contributions

JD and DS: conceptualization and supervision. LM and MG: data curation, formal analysis, investigation, and methodology. ZC: project administration. LM, YY, and PC: software and writing (original draft). MG and DS: writing (review and editing). LM, DP, and JG: modified the final version. All authors contributed to the article and approved the submitted version.

## Funding

This work was supported by the project of Major New Drug Creation (No. 2018ZX09301-011-001), National Natural Science Foundation of China (Grant No. 81904025, 81904046), and Fundamental Research Funds for the Central Public Welfare Research Institutes Grant (Grant No. ZZ13-YQ-016 and ZZ13-YQ-016-C1).

## Conflict of Interest

The authors declare that the research was conducted in the absence of any commercial or financial relationships that could be construed as a potential conflict of interest.

## Publisher's Note

All claims expressed in this article are solely those of the authors and do not necessarily represent those of their affiliated organizations, or those of the publisher, the editors and the reviewers. Any product that may be evaluated in this article, or claim that may be made by its manufacturer, is not guaranteed or endorsed by the publisher.
