# Supplementary material for: Serum Uric Acid and Risk of Chronic Heart Failure: A Systematic Review and Meta-Analysis
Source: Front Med (Lausanne). 2021 Dec 14;8:785327. doi: 10.3389/fmed.2021.785327 (PMC8715937; doi:10.3389/fmed.2021.785327)
Supplement: Supplementary file 1 [file Data_Sheet_1.PDF]

Pubmed search terms

(("Heart Failure"[Mesh]) OR (((((((((((((((Heart Failure[Title/Abstract]) OR (Cardiac Failure[Title/Abstract])) OR (Heart Decompensation[Title/Abstract])) OR (Decompensation, Heart[Title/Abstract])) OR (Heart Failure, Right-Sided[Title/Abstract])) OR (Heart Failure, Right Sided[Title/Abstract])) OR (Right-Sided Heart Failure[Title/Abstract])) OR (Right Sided Heart Failure[Title/Abstract])) OR (Myocardial Failure[Title/Abstract])) OR (Congestive Heart Failure[Title/Abstract])) OR (Heart Failure, Congestive[Title/Abstract])) OR (Heart Failure, Left-Sided[Title/Abstract])) OR (Heart Failure, Left Sided[Title/Abstract])) OR (Left-Sided Heart Failure[Title/Abstract])) OR (Left Sided Heart Failure[Title/Abstract]))) AND (("Uric Acid"[Mesh]) OR ((((((((((((((((((((((((Uric Acid[Title/Abstract]) OR (Acid, Uric[Title/Abstract])) OR (2,6,8-Trihydroxypurine[Title/Abstract])) OR (Trioxopurine[Title/Abstract])) OR (Potassium Urate[Title/Abstract])) OR (Urate, Potassium[Title/Abstract])) OR (Urate[Title/Abstract])) OR (Ammonium Acid Urate[Title/Abstract])) OR (Acid Urate, Ammonium[Title/Abstract])) OR (Urate, Ammonium Acid[Title/Abstract])) OR (Sodium Urate Monohydrate[Title/Abstract])) OR (Monohydrate, Sodium Urate[Title/Abstract])) OR (Urate Monohydrate, Sodium[Title/Abstract])) OR (Monosodium Urate Monohydrate[Title/Abstract])) OR (Monohydrate, Monosodium Urate[Title/Abstract])) OR (Urate Monohydrate, Monosodium[Title/Abstract])) OR (Sodium Acid Urate Monohydrate[Title/Abstract])) OR (Sodium Urate[Title/Abstract])) OR (Urate, Sodium[Title/Abstract])) OR (Monosodium Urate[Title/Abstract])) OR (Urate, Monosodium[Title/Abstract])) OR (Sodium Acid Urate[Title/Abstract])) OR (Acid Urate, Sodium[Title/Abstract])) OR (Urate, Sodium Acid[Title/Abstract])))

Embase search terms

#42. #25 AND #41
#41. #26 OR #27 OR #28 OR #29 OR #30 OR #31 OR #32 OR #33 OR #34 OR #35 OR #36 OR #37 OR #38 OR #39 OR #40
#40. 'left sided heart failure':ti,ab
#39. 'left-sided heart failure':ti,ab
#38. 'heart failure, left sided':ti,ab
#37. 'heart failure, left-sided':ti,ab
#36. 'heart failure, congestive':ti,ab
#35. 'congestive heart failure':ti,ab
#34. 'myocardial failure':ti,ab
#33. 'right sided heart failure':ti,ab
#32. 'right-sided heart failure':ti,ab
#31. 'heart failure, right sided':ti,ab
#30. 'heart failure, right-sided':ti,ab
#29. 'decompensation, heart':ti,ab
#28. 'heart decompensation':ti,ab
#27. 'cardiac failure':ti,ab
#26. 'heart failure'/exp
#25. #1 OR #2 OR #3 OR #4 OR #5 OR #6 OR #7 OR #8 OR

#9 OR #10 OR #11 OR #12 OR #13 OR #14 OR #15 OR
#16 OR #17 OR #18 OR #19 OR #20 OR #21 OR #22 OR
#23 OR #24

#24. 'urate, sodium acid':ti,ab
#23. 'acid urate, sodium':ti,ab
#22. 'sodium acid urate':ti,ab
#21. 'urate, monosodium':ti,ab
#20. 'monosodium urate':ti,ab
#19. 'urate, sodium':ti,ab
#18. 'sodium urate':ti,ab
#17. 'sodium acid urate monohydrate':ti,ab
#16. 'urate monohydrate, monosodium':ti,ab
#15. 'monohydrate, monosodium urate':ti,ab
#14. 'monosodium urate monohydrate':ti,ab
#13. 'urate monohydrate, sodium':ti,ab
#12. 'monohydrate, sodium urate':ti,ab
#11. 'sodium urate monohydrate':ti,ab
#10. 'urate, ammonium acid':ti,ab
#9. 'acid urate, ammonium':ti,ab
#8. 'ammonium acid urate':ti,ab
#7. 'urate':ti,ab
#6. 'urate, potassium':ti,ab
#5. 'potassium urate':ti,ab
#4. 'trioxopurine':ti,ab
#3. '2,6,8-trihydroxypurine':ti,ab
#2. 'acid, uric':ti,ab
#1. 'uric acid'/exp
